# Bone-Regenerating Capacity of Chitosan Membrane and Chitosan Foam Scaffolds in Critical Size Defects: In Vitro and In Vivo Study

**DOI:** 10.3390/dj13040153

**Published:** 2025-03-31

**Authors:** Iman A. Fathy, Dina M. Ali, Youssef Elmansy, Nour E. A. Abd El-Sattar, Sherif Elsayed

**Affiliations:** 1 Oral Biology Department, Faculty of Dentistry, Ain Shams University, Cairo 11566, Egypt; iman.fathy@dent.asu.edu.eg; 2 Botany Department, Faculty of Science, Ain Shams University, Cairo 11566, Egypt; dinamohamedali12@sci.asu.edu.eg; 3 Military Medical Academy, Cairo 11766, Egypt; elmansi76@gmail.com; 4 Department of Chemistry, Faculty of Science, Ain Shams University, Cairo 11566, Egypt; nourel-dinahmed@sci.asu.edu.eg; 5 Basic & Medical Sciences Department, Faculty of Dentistry, Al-Ryada University for Science & Technology, Sadat City 5730306, Egypt; 6 Department of Prosthodontics, Faculty of Dentistry, Al-Ryada University for Science and Technology, Sadat City 5730306, Egypt

**Keywords:** chitosan, bone regeneration, tissue engineering, bone scaffold, freeze drying, scaffold porosity, chitosan foam, chitosan membrane

## Abstract

**Objectives**: The objective of this study is to compare the bone-regenerating capacity between chitosan foam and chitosan membrane scaffolds. **Methods**: A medium-weight chitosan acidic mixture was used to prepare two scaffolds of freeze-dried chitosan foam (CF). One of the two CF scaffolds was physically crosslinked by NaHCO_3_ to obtain chitosan membrane (CM). A morphological assessment of the specimens’ porosity was carried out by scanning electron microscopy (SEM). An MTT assay of the CM and CF specimens using rats’ bone marrow mesenchymal stem cells (MSCs) was carried out. Then, 38 albino rats were subjected to surgical implantation in a critical-size defect of the femur bone. The rats were divided into three groups according to the type of implanted scaffold (Control (no scaffold) *n* = 10, CM (chitosan membrane) *n* = 14, CF (chitosan foam) *n* = 14). Each group was equally subdivided into two subgroups according to the time of euthanasia (21 d, 35 d). The femur bones were dissected for a histological analysis (hematoxylin and eosin, and Masson trichrome). The results of the histological analysis were graded according to a scoring system. A statistical analysis of the pore size and histological grading was carried out. **Results**: CF had a higher mean pore size (65.42 µm) compared to CM (6.44 µm); CM showed a significantly higher proliferation of MSCs at 72 h. Both the CM and CF groups showed a significantly higher bone regeneration and lower inflammation than the control group. The CF group showed a significantly higher bone regeneration score than the CM group, especially at 35 d with more dense compact lamellar bone structure. **Conclusions**: The higher mean pore size of CF allowed for a higher bone regenerating capacity than the crosslinked CM.

## 1. Introduction

Bone tissues have a unique ability to heal and regenerate after damage. Bone fractures, in several cases, can be immobilized to give them a chance to spontaneously heal with time. But, if bone defects were large enough to reach what is known as a critical-size defect, it would be difficult for these large defects to heal by a physiological process, and they would require interventions in the form of bone grafts [1].

Autogenous and allogenic bone grafts are commonly used for critical-size bone defects. However, their use has some disadvantages, such as donor-site morbidity, cross-infections, and ethical concerns about using allografts [2]. Tissue engineering (TE) involves using a three-dimensional (3D) scaffold to create a suitable environment, which is conductive to cells and growth factors, helping with the regeneration of impaired tissues [3].

Bone tissue engineering aims to replicate the complicated structures of compact and cancellous bones in order to induce bone regeneration and integration [4]. The porosity and pore size of bone tissues have great effects on the function and integrity of tissues. Compact bone porosity is mainly related to the Haversian system, with canals ranging from about 20 to 100 μm in diameter—these canals are interconnected by Volkmann’s canals [3]. Cancellous (spongy) bone forms the inner layer of bone—it is crucial for the metabolic activity of the tissue. This spongy layer contains a network of trabeculae, that are interconnected, and provides structural support for bone, and, at the same time, increases the adaptability of bones to mechanical forces. The porous structure of spongy bone enables it to include blood vessels and bone marrow, leading to an efficient nutrient supply to bone tissue [4].

Bone tissue engineering (BTE) involves the interplay between three critical components: cells, scaffold, and signaling factors. Scaffolds can be considered as a temporary extracellular matrix for bone regeneration. Several properties of scaffolds are critical for the determination of the bone regeneration fate, such as biocompatibility, osteoconductivity, osteoinductivity, osteogenic ability, mechanical properties, porosity, and pore size and morphology [5]. Pore size, distribution, and morphology, in particular, are critical determinants of the bone regeneration fate—they are considered as knobs for bone-healing biological reactions [6].

The deacetylation of chitin produces chitosan; chitosan is a natural polymer with β−(1 → 4)-glycosidic bond linking (1 → 4)-2-amino-2-deoxy-D-glucan (D-glucosamine). Chitosan can be extracted from the shells of crustaceans such as shrimps, fish scales, insects, or fungi. Recently, due to the high cost of this extraction, other sources for chitosan are under investigation, such as food waste [7].

Chitosan can be fabricated by different methods to produce 3D porous BTE scaffolds, with promising results in the literature [8]. The freeze-drying (FD) technique of scaffold fabrication depends on the sublimation process, to produce a highly porous scaffold with a high surface-area-to-volume ratio. The FD fabrication process for fabricating chitosan scaffolds includes three steps: (I) the preparation of the chitosan mixture, e.g., by dissolving in diluted acetic acid; (II) freezing; and (III) the lyophilization of the frozen solvent. Sublimation removes the solvent from the frozen chitosan mixture. The porosity of chitosan scaffolds can be tailored, by controlling the concentration of the chitosan solution, duration, and rate of freezing [8,9].

In the current study, two forms of chitosan scaffolds fabricated by FD (chitosan foam and chitosan membrane) were investigated, regarding their porosity, cytotoxicity, and bone-regenerating capacity in critical defects of rats’ femur bones. The null hypothesis assumed in this study was that there was no significant difference between the two forms of chitosan scaffolds regarding their bone regeneration capacity.

## 2. Materials and Methods

### 2.1. Preparation of Scaffolds

- 1.14 gm of chitosan powder (90–95% deacetylation degree—medium molecular weight—ChitoLab—Egypt) was dissolved in 100 mL of 2% acetic acid, and mixture was magnetic stirred for 2 h at 500–600 rpm. After stirring, mixture was injected using sterile plastic syringe into 2 sterile plastic cups (14 mL in each cup).
- Chitosan solution in the two cups was frozen at −80 °C for 24 h, then freeze-dried in lyophilizer (CHRIST Alpha 1–4 LD plus (serial number: 22695)) for 48 h. Parameters of lyophilization were as following: freezing (15 min), then main drying (temperature of −45 °C, pressure was 0.070 mbar, and time was 2 days), and final drying stage (−76 °C, 0.0010 mbar, and 15 min).

One of the two chitosan foam (CF) specimens that were produced by freeze-drying was immersed in 0.2 M sodium bicarbonate solution for about 90 min for physical crosslinking, to produce crosslinked chitosan membrane (CM). Finally, CM was washed several times by double-distilled water.

### 2.2. Morphological Evaluation of Scaffolds by Scanning Electron Microscopy

#### 2.2.1. Scanning Electron Microscopy (SEM) Imaging

Either chitosan foam (CF) or chitosan membrane (CM) specimen was cut into small pieces (2 × 1 mm), and one piece from each group (CF and CM) was subjected to scanning electron microscope scanning, using field emission microscope (FESEM QUANTA FEG 250, Eindhoven, The Netherlands), for morphological assessment.

#### 2.2.2. Pore Size Measurement

Pore configurations and dimensions in each specimen from each group were evaluated and measured using field emission microscope, regarding pore size, distribution, and shape. Pore measurements from scaffolds of CM and CF were subjected to statistical analysis, by taking 30–50 measurements from each SEM photomicrograph, with a total of 3 images (140–150 measurements) for each group (CM and CF). Pore measurements analysis was carried out using open-source image analysis software (Imagej 1.54 g NIH, Bethesda, MD, USA).

Statistical analysis of measurements was performed with SPSS 20^®^, Graph Pad Prism^®^, and Microsoft Excel 2016. All numerical data were presented as mean and standard deviation. Normality of data was tested by using Kolmogorov and Shapiro–Wilk’s test, while homogeneity of data was assessed using Levene’s test.

### 2.3. Biocompatibility Assessment of Mesenchymal Stem Cells

#### 2.3.1. Mesenchymal Stem Cell (MSC) Isolation and Subculturing

Three albino rats were dissected to obtain intact femurs and tibiae bones. Bones were transferred to Biosafety cabinet in transfer media (Dulbecco’s Modified Eagle’s Medium (DMEM) + antibiotic/antifungal).

Inside Biosafety cabinet (Thermo Scientific^TM^ Thermo Safety Bench Msc Advantage 1.2-LN.51025411-S.N, Ontario, Canada), heads of bones were removed using a sterile scissor. Bone marrow was aspirated with sterile needle and resuspended in culture media (75 mL DMEM, 15 mL fetal bovine serum (FBS), 5 mL penicillin/streptomycin mixture, and 50 microgram antifungal) in T 75 culturing flasks. Cell cultures were obtained in the final complement culture media. The culture flasks were incubated in a humidified incubator (Thermo-Scientific-Midi-40-CO_2_-incubator-I.N.3404-S.N.300383414) at 37 °C in 5% CO_2_ and 95% air by volume. The cultured cells were examined daily by using the inverted microscope (Leica DM IL LED Inverted Fluorescence Microscope, Wetzlar, Germany) to follow up the growth of the cells and to detect the appearance of any bacterial or fungal infection among the cultured cells. After 48 h, the supernatant that contained the non-adherent cells was removed by aspiration using a sterile pipette. The adherent cells were then washed twice with a sterile PBS and 10 mL of fresh complete media was added to the dish. Hence, MSCs were distinguished from other cells by their tendency to adhere to tissue culture plastic. The exchange of media was carried out day after day.

#### 2.3.2. Characterization of MSCs

Isolated rats’ bone marrow MSCs were examined using a FACS Canto II Flow Cytometer with a 488 nm argon laser. Cells were treated for 60 min at 4 °C with fluorescein isothiocyanate-conjugated monoclonal antibodies targeting rat CD45 (Clone REA504), rat CD90 (Clone REA838), and rat CD105 (Clone REA683), all sourced from Miltenyi Biotec GmbH. Isotype-identical antibodies functioned as controls. Representative samples were examined by acquiring 10,000 events on the FACS Canto II Flow Cytometer utilizing BDFACS Diva™ v6.1.3 software.

**Figure 1:**
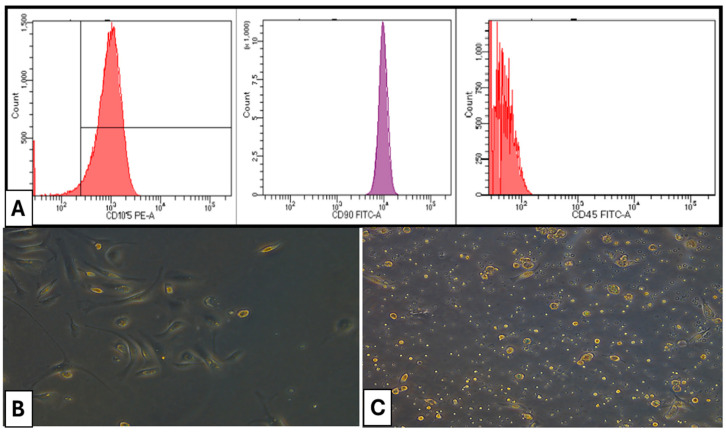
(**A**) Flow cytometry characterization of isolated cells. (**B**) Photomicrographs of a culture of rat bone marrow MSCs (×100) on day 7 of culture showing fibroblast-like cells (**C**) Photomicrographs of a culture of rat bone marrow MSCs (×100) on day 14 of culture showing confluent cells exhibiting various morphologies.

CD105 and CD90 of bone marrow MSCs exhibited positivity at 98.4% and 98.7%, respectively. Conversely, the presence of CD45 in hematopoietic cells was seen at merely 0.7%, indicating that the separated bone marrow MSCs were homogeneous and exhibited a high purity of mesenchymal stem cells (Figure 1).

#### 2.3.3. Cytotoxicity Evaluation by MTT Assay

Specimens from CM and CF scaffolds were cut into small pieces to be incubated with cell culture media (DMEM) in 96-well plate, for 24 h at temperature of 37 °C in an incubator with 5% CO_2_.

Isolated rats’ bone marrow MSCs were inoculated dropwise with concentration of 1 × 10^5^ cells/mL (100 uL/well) in 96-well plate, where cells were allowed to set on pieces of tested scaffolds for different time intervals according to study design (24 h (T1), 48 h (T2), and 72 h (T3)), and incubated at 37 °C and with 5% CO_2_ to develop a complete monolayer sheet.

At established times, media were removed and replaced by standard media alone. Plate was incubated at 37 °C and examined. Cells were checked for any physical signs of toxicity, e.g., partial, or complete loss of the monolayer, rounding, shrinkage, or cell granulation. A 3-(4,5-dimethylthiazol-2-yl)-2,5-diphenyl tetrazolium bromide (MTT) assay solution was prepared (5 mg/mL in PBS) (R&D systems: #4890-25-01). A 20 μL MTT solution was added to each well and the MTT was thoroughly mixed into the media, followed by incubation (37 °C, 5% CO_2_) for 3 h to allow the MTT to be metabolized. Formazan (MTT metabolic product) was resuspended in 20 μL/well DMSO, and the formazan was mixed thoroughly into the solvent. Optical density at 560 nm was read by spectrophotometer and subtracted from background at 620 nm, using Thermo ScientificTM, Waltham, MA, USA, MultiskanTM Sky Microplate Spectrophotometer [10]. Three specimens were examined for each treatment group, positive control group (only cells in media without scaffolds) and negative control group (without cells) at T1, T2, and T3, and experiment was repeated for each group in triplicates.

Statistical analysis of optical density (OD) was performed with SPSS 20^®^, Graph Pad Prism^®^, and Microsoft Excel 2016. All numerical data were presented as mean and standard deviation. Normality of data was tested by using Kolmogorov and Shapiro–Wilk’s test, while homogeneity of data was assessed using Levene’s test.

### 2.4. Power Analysis of Sample Size for Animal Groups

Sample size calculation of animal groups for in vivo application of scaffolds was performed using G*Power version 3.1.9.7 based on the results of a previous study [11]. Power analysis was designed to have adequate power to apply a two-sided statistical test to reject the null hypothesis that there was no difference between groups, by adopting an alpha level of (0.05) and a beta of (0.2), i.e., power = 85% and an effect size (d) of (0.9) calculated based on the results of a previous study. The predicted sample size is 7 per treatment group (*n* = 7).

### 2.5. In Vivo Application of Scaffolds

#### 2.5.1. Presurgical Preparation of Scaffolds

**Figure 2:**
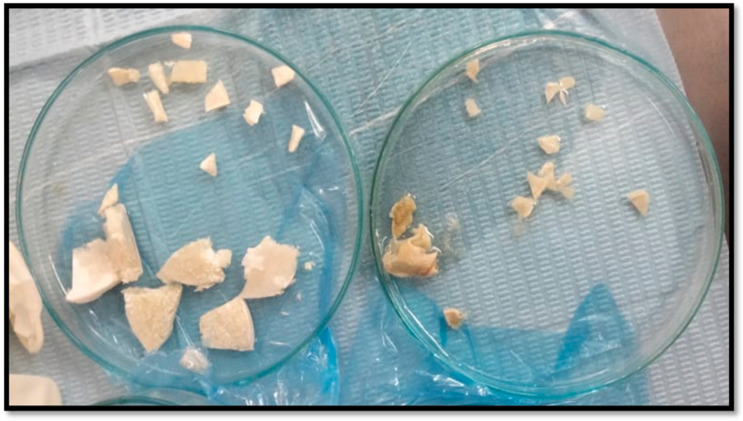
Cutting chitosan pieces before surgical insertion: chitosan foam (**left**), and chitosan membrane (**right**).

CM and CF scaffolds were cut into small pieces using sterile lancet to fit into surgical defects (Figure 2). Then, scaffolds pieces were washed by sterile phosphate-buffered saline (PBS) several times. Afterwards, scaffold pieces from the two groups were disinfected by UV irradiation for 60 min [12]. Scaffolds pieces were finally disinfected by spraying with ethyl alcohol 70% and left to dry, directly before implantation in rats at the operating room.

#### 2.5.2. Animal Grouping and Study Design

The experimental animals were 38 adult female albino rats (90 d old) that weighed an average of 150 g (130–170 g) at the beginning of the study. During the period of the experiment, the animals were kept in plastic cages with access to food and drinking water ad libitum, and a fat-rich diet was presented to all animals for 2 months until random blood sugar of 150–200 mg/dL was obtained and serum cholesterol reached 113 mg/dL ± 2 in 80% of animals. The study design and surgical protocol were approved by the Animal Care and Use and Ethical Committee of Faculty of Dentistry, Ain Shams University [FDASU-Rec IR022535].

The other three rats were subjected to euthanasia for isolation of mesenchymal stem cells as previously mentioned. The experimental 38 rats were divided into 3 groups according to the type of scaffold used, as follows: control group, CM groups, and CF groups. Control group included ten animals (*n* = 10), while each treatment group included fourteen animals (*n* = 14). Animals in each group were equally subdivided into two subgroups according to the time of euthanasia (21 d and 35 d). In control subgroups, the surgical defects of rats’ femur bones were left empty without scaffold, while, in CM subgroups, surgical defects were implanted by chitosan membrane scaffolds, and, in CF subgroups, surgical defects were implanted by chitosan foam scaffolds.

#### 2.5.3. Surgical Procedures

After a week of acclimatization, rats of different groups were anesthetized by intraperitoneal injection of Ketamine Hydrochloride^®^ 10% (35 mg/kg body weight) and Xylazine Hydrochloride^®^ 2% (5 mg/kg body weight) [13]. Following anesthesia, the surgical site (dorsal surface) was shaved and disinfected with Betadine^®^ [14].

**Figure 3:**
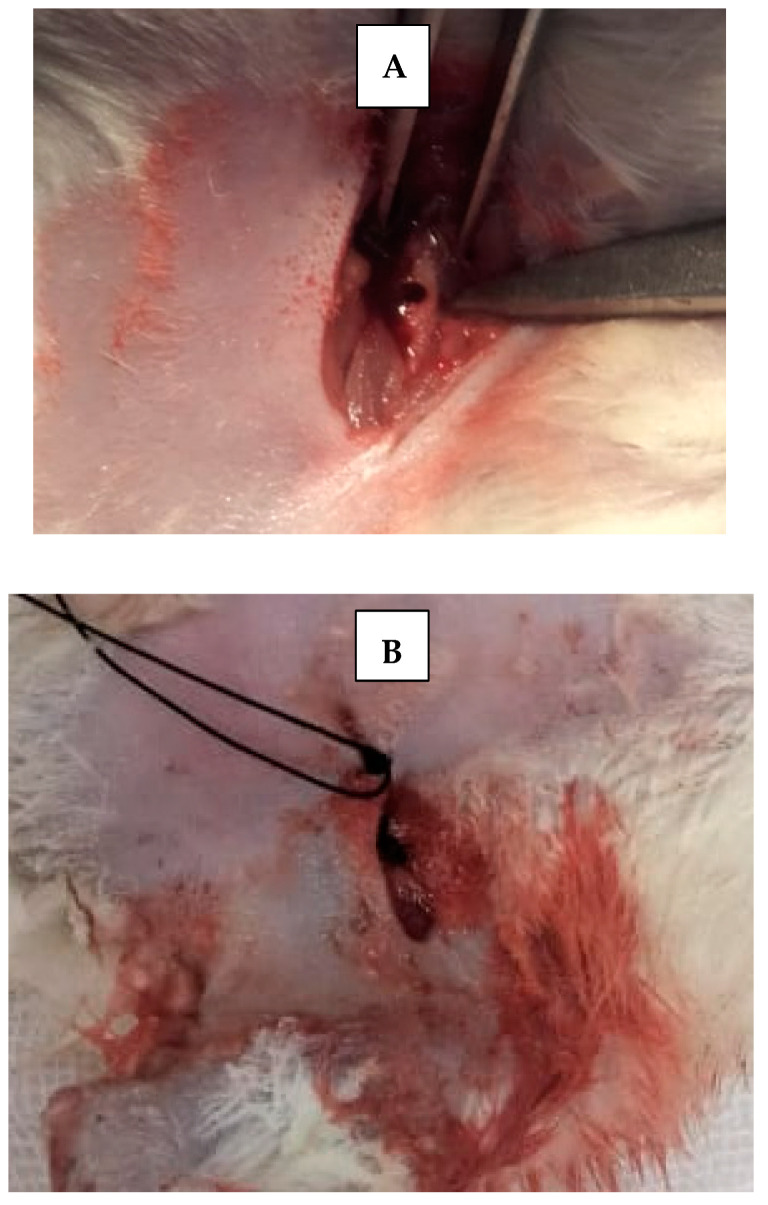
(**A**) Critical size surgical defect in rat’s femur bone. (**B**) Suturing with non-resorbable suture.

Skin incisions were made, and the periosteum was retracted using a fine elevator to expose femur bone. A 5 mm critical bone defect [15] was created in the upper part of each femur bone of each rat, using straight microsurgical handpiece (Figure 3), accompanied by irrigation with cold sterile saline solution, to prevent bone necrosis by heat and to constantly clean surgical field from bone debris. Then, scaffolds were applied inside created bone defects using fine pluggers according to the rat treatment group. Surgical defects were left empty without scaffold in control groups. The skin was sutured with 4–0 nonabsorbable sutures. Afterwards, rats were transferred to a warm recovery room [13].

#### 2.5.4. Histological and Histochemical Assessment

At the end of the experimental period of each subgroup, rats were examined clinically, and healing complications were noticed only in positive control subgroups. Rats were subjected to euthanasia by an overdose of anesthesia, and, then, femurs were retrieved and freed from any soft tissue carefully. The rest of rats’ bodies were appropriately gotten rid of in the incinerator of Ain Shams Teaching Hospital.

##### Specimen Retrieval, Histological Staining, and Histochemical Staining

The lower femur bones were dissected, and the specimens were fixed in 4% neutral formalin for 48 h and subsequently demineralized in a solution containing equal parts of 50% formic acid and 20% sodium citrate for 30 days. Once demineralization was achieved and checked by sharp probe, specimens were processed, and paraffin serial cross-sections (4 μm) were obtained. Some of these sections were stained by hematoxylin and eosin, while others were stained by Masson trichrome. Histological scoring data were collected and tabulated for statistical analysis.

##### Qualitative Histopathological Scoring

For both H&E and Masson trichrome stain photomicrographs, images were first corrected for brightness and contrast. Two sections from each sample were examined and graded by an expert pathologist for bone regeneration (grades 1–3), assessing inflammation (grades 0–3), fibrotic reaction (+/−), and bone vitality (1/0). Bone regeneration was graded 1 if woven bone was observed, 2 if both woven and lamellar bones were observed, and 3 if just lamellar bone was observed. Inflammation was graded as based on the number of infiltrative cells seen in a visual field, from 0 to 3. The decision on fibrotic tissue reaction was made based on the presence of dense fibrous areas. Bone vitality was signed as positive (1) if there were osteocytes inside lacunae. Figure plates containing original images were compiled to compare the histological variations [13].

Statistical analysis of the histological and histochemical results of all subgroups was performed using SPSS (IBM SPSS Statistics 21.0, IBM Corporation, and Somers, NY, USA). Comparison between groups was carried out using one-way analysis of variance (ANOVA) followed by a post hoc test. Numerical data of groups were also analyzed (a *p*-value < 0.05 was selected as significant). Values were determined as means ± standard deviation.

## 3. Results

### 3.1. Morphological Evaluation by Scanning Electron Microscopy

#### 3.1.1. Scanning Electron Microscopy Imaging

**Figure 4:**
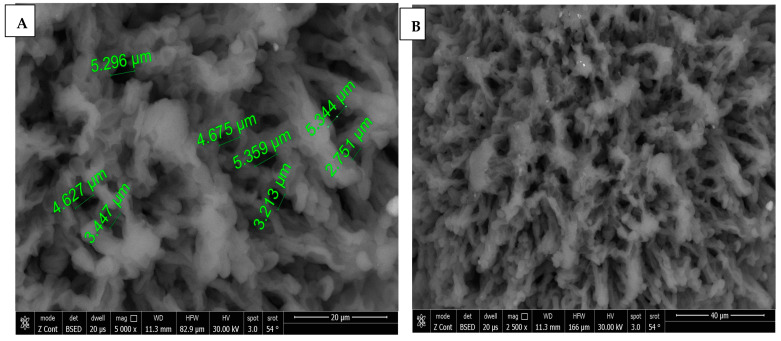

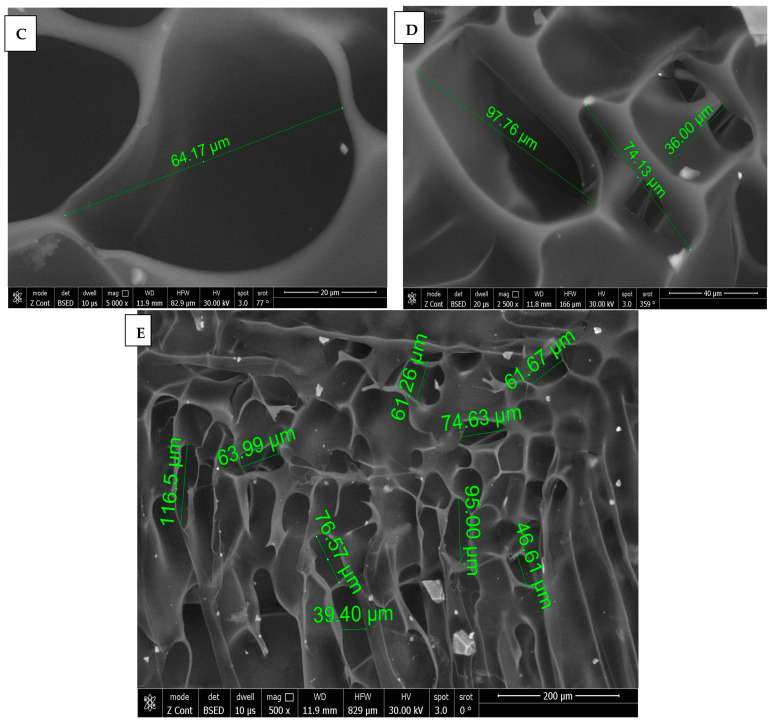
(**A**) 5000× SEM photomicrograph image of chitosan membrane (CM) with measurements of pores using digital ruler. (**B**) 2500× SEM photomicrograph image of chitosan membrane (CM) showing narrow pores between crosslinked polymer chains. (**C**,**D**) Showing 5000× and 2500× SEM photomicrograph images of chitosan foam (CF), respectively, with digital ruler measurements of pores. (**E**) 500× SEM photomicrograph image of chitosan foam (CF) showing wide elliptical-shaped pores with digital measurements.

SEM images of chitosan membrane (CM) with 5000× and 2500× magnifications were illustrated in Figure 4A,B, which demonstrated the irregular distribution of narrow-sized pores, and the collapsing of chitosan chains was evident due to the effect of crosslinking [16], while, in Figure 4C–E, SEM images of chitosan foam (CF) (5000×, 2500×, 500×) illustrated a more regular distribution of elliptical-shaped wide-sized pores.

#### 3.1.2. Pore Size Measurements

Normality tests showed the pore size measurements were non-parametric. Accordingly, a comparison between CM and CF was performed by using a Mann–Whitney test. 

**Figure 5:**
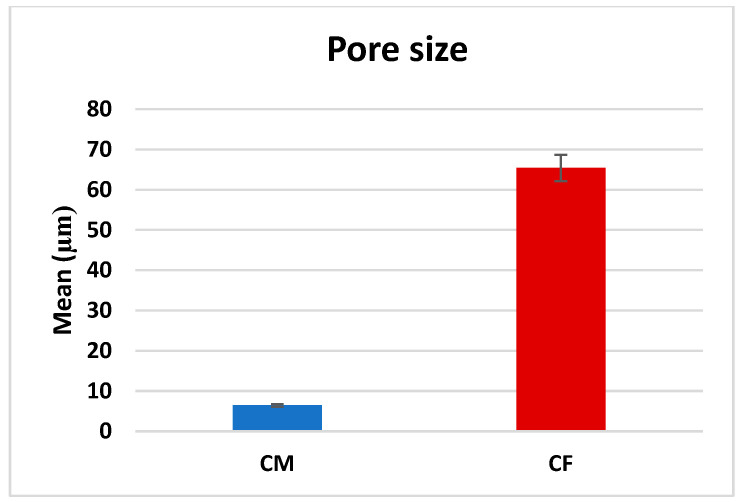
Bar chart representing mean pore size measurements in CM and CF scaffolds.

A comparison between CF and CM regarding the mean pore size revealed that there was a significant difference (*p* < 0.001), as CF (65.42 ± 31.13 µm) was significantly higher than CM (6.44 ± 2.78 µm), as presented in Table 1 and Figure 5.

### 3.2. Biocompatibility Assessment of Mesenchymal Stem Cells

#### Cytotoxicity Evaluation by MTT Assay

**Figure 6:**
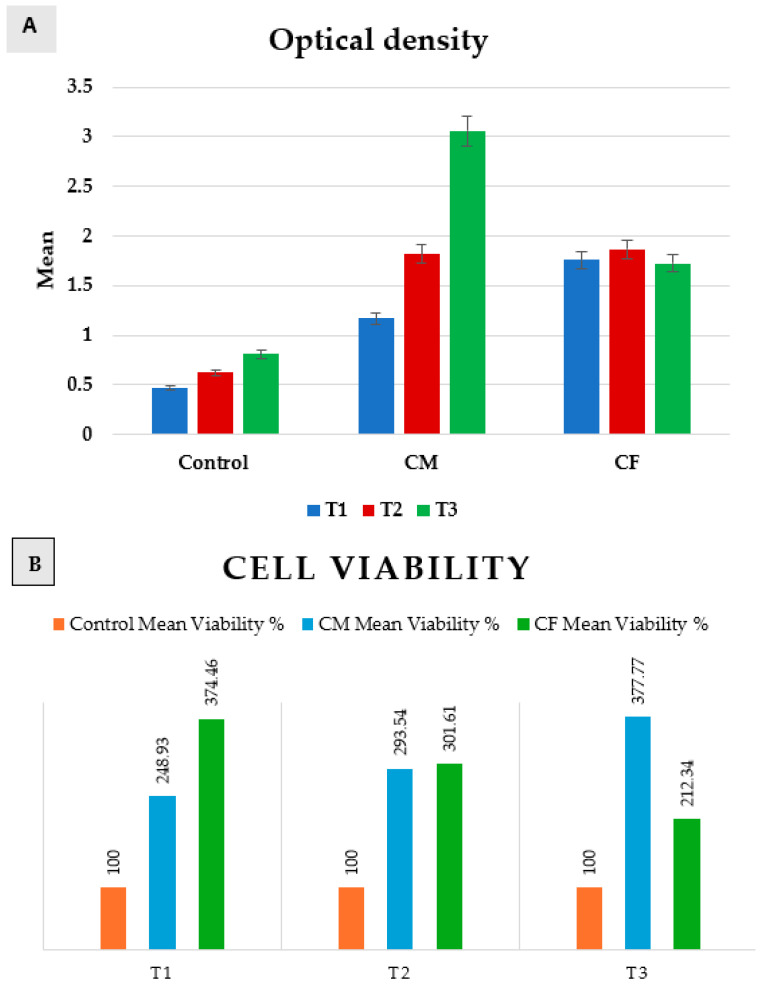
Bar chart representing both (**A**) optical density, and (**B**) cell viability in positive control group, CM, and CF at T1, T2, and T3.

Measurements of optical density revealed a normal data distribution. Accordingly, a comparison between positive control, CM, and CF was performed by using a one-way ANOVA test, followed by Tukey’s post hoc test, while a comparison between T1, T2, and T3 was performed by using a repeated-measures ANOVA test, followed by Tukey’s post hoc test for pairwise comparisons (Table 2 and Figure 6).

A comparison between groups revealed that there was a significant difference between them at *p* = 0.0001 at T1, T2, and T3.

In **T1** (24 h), the positive control group was significantly the least, and then CM, while CF was significantly the highest. In **T2** (48 h), control was significantly the least, while there was an insignificant difference between CM and CF. In **T3** (72 h), the control group was significantly the least, and then the CF group, while CM was significantly the highest.

Regarding CM, a comparison between T1, T2, and T3 revealed that there was a significant increase from (1.17 ± 0.12) at T1, to (1.82 ± 0.02) at T2, and then a significantly increase to (3.06 ± 0.2) at T3 as *p* = 0.007. But, in the control group and CF, there was an insignificant change between T1, T2, and T3 as *p* = 0.06 and 0.29, respectively.

Scaffolds of both groups proved to be non-cytotoxic on rats’ bone marrow MSCs, as the cultured cells on scaffolds showed high proliferation rates (>100%) at 24 h, 48 h, and 72 h when compared to the positive control group. The results revealed that the highest proliferation rate was after 72 h of incubation in DMEM for the CM group.

### 3.3. In Vivo Application of Scaffolds

#### 3.3.1. Histological and Histochemical Assessment

One rat from the control group showed signs of impaired wound healing and infection. There was no bacterial deep wound infection in any of the rats that underwent the procedure. No rat died after the end of the experiment.

##### Three-Week (21 d) Group—“Hematoxylin and Eosin Staining”

**Figure 7:**
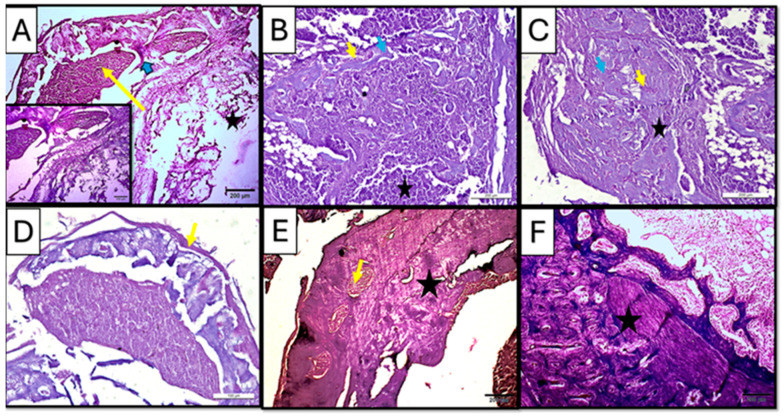
Photomicrographs of hematoxylin-and-eosin-stained sections of the defect in rat’s femur bone. (**A**) Control subgroup at 21 d, showing granulation tissue formation (yellow arrow), fat cells in bone marrow (star), and spicules of woven bone (blue arrow). Insert: magnification of defect area showing irregularity of the formed bone tissue and densely packed inflammatory cells in the granulation tissue. (**B**) CM subgroup at 21 d, showing infiltration with inflammatory cells (star) besides the appearance of osteoprogenitor cells/osteoblasts rimming the marrow cavities (yellow arrow); viable osteocytes were also noticed (blue arrow). (**C**) CF subgroup at 21, d showing organized collagen fibers (star) besides the appearance of osteoprogenitor cells/osteoblasts rimming the marrow cavities (yellow arrow); viable osteocytes were also noticed (blue arrow). (**D**) Control subgroup at 35 d, showing a fibrous organized periosteum (yellow arrow) encapsulating dome-shaped tissue was observed. (**E**) CM subgroup at 35 d, showing bone trabeculae (star) were detected with proper vascularity (yellow arrow) visible in the marrow cavity. (**F**) CF subgroup at 35 days, showing lamellar bone and distinct osteons (star) (H&E ×100).

Regular hematoxylin and eosin staining showed newly formed trabecular bone in both treated subgroups (CF and CM) of a three-week interval, suggesting active bone remodeling (Figure 7B,C). However, the control subgroup showed sparse marrow cavities and osteoid formation in some areas, while fatty bone marrow was observed (Figure 7A).

Early to intermediate stages of bone formation were observed in the CM subgroup at 3 weeks with dense connective tissue representing fibrous tissue or areas of pre-bone formation. Moreover, active cellular activity, including dense infiltration with inflammatory cells besides the appearance of osteoprogenitor cells/osteoblasts rimming the marrow cavities, was observed; viable osteocytes were also noticed (Figure 7B), while the CF subgroup at the same duration of 3 weeks showed organized collagen fibers, forming concentric patterns in large, rounded areas likely corresponding to osteons or newly mineralized regions. Such findings suggested an early lamellar structure indicative of advancing regeneration in the CF subgroup (Figure 7C).

##### Five-Week (35 d) Period—“Hematoxylin and Eosin Staining”

Regular hematoxylin and eosin staining in the control subgroup showed, after 5 weeks of scaffold implementation, a dome-like structure encapsulating bone tissue, with an organized periosteum being observed, indicating an attempt to functionally regenerate the lost structures in a rather non-anatomical manner (Figure 7D).

On the other hand, the treated subgroups showed mature bone formation, which was evident with compact, organized regions. In the CM subgroup, mature bone trabeculae were detected with proper vascularity visible in the marrow cavity, suggesting ongoing remodeling and vascularization (Figure 7E).

In CF subgroup, however, lamellar bone and distinct osteons could be seen in all specimens. Moreover, the presence of mature periosteal and endosteal layers indicated well-organized new bone regeneration in the CF subgroup (Figure 7F).

**Masson trichrome staining:**

Masson trichrome staining at a three-week interval revealed the presence of newly formed bone matrix (blue stain) collectively in all subgroups, where the progressive stages of bone healing were observed starting from early fibrous tissue formation in control and CM to organized lamellar bone in the CF subgroup. Newly formed tissue was represented in the CM subgroup as organized collagen fibers, forming concentric patterns in the CF subgroup resembling early osteoid or woven bone.

**Figure 8:**
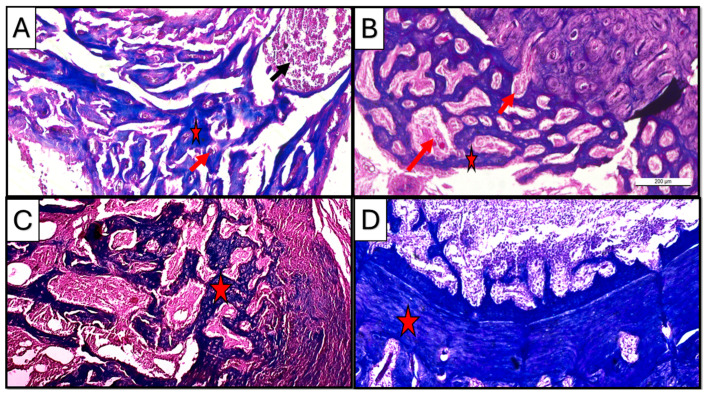
Photomicrographs of Masson-trichrome-stained sections of the defect in rat’s femur bone. (**A**) CM subgroup at 21 d, showing spicules of woven bone (star), inflammatory cells in bone marrow (black arrow), and blood vessels inside newly formed tissue (red arrow). (**B**) CF subgroup at 21 d, showing regular newly formed bone trabeculae (star) with rich blood supply (arrow). (**C**) CM subgroup at 35 d, showing regularly formed lamellar bone trabeculae with well-vascularized marrow cavities (star). (**D**) CF subgroup at 35 d, showing densely packed regenerated collagen fibers forming a compact bone layer (star) (Masson trichrome ×100).

However, at a five-week interval, mature bone with organized lamellar structures was observed in all subgroups, specifically in treated groups where newly regenerated tissues showed organized trabecular pattern in the CM subgroup, while the CF subgroup showed more dense compact bone structure (Figure 8).

Regarding vascularity, copious neovascularization activity was observed in the CF group as compared to the CM group, especially in the three-week duration at the early to intermediate bone regeneration phases (Figure 8A,B).

#### 3.3.2. Qualitative Histopathological Scoring

The histological scoring results were illustrated in Table 3. At three weeks, the CF subgroup showed the significantly lowest mean inflammation (1.00 ± 0.71) and the lowest median inflammation, indicating better results compared to the control and CM subgroups, while, in the fifth week, the CF subgroup again showed the best results with a mean of 0.00 ± 0.00, indicating no inflammation, while the control subgroup had a mean of 2.20 ± 0.45 and the CM subgroup had 1.20 ± 0.84.

At a three-week interval, the CF subgroup had a higher percentage of absent fibrosis (60.0%) compared to the control subgroup (0.0%) and CM subgroup (40.0%).

At a five-week interval, both the CM and CF subgroups showed no presence of fibrosis (0.0%), while the control subgroup had a 60.0% presence of fibrosis with a significant difference, indicating that both the CM and CF subgroups had better healing outcomes [13].

Related to bone vitality, all groups (Control, CM, and CF) showed a 100% presence of vital osteocytes, indicating no significant differences among the groups on both durations of three weeks and five weeks.

The CF group showed the significantly highest mean bone regeneration (2.20 ± 0.45) and median (2) after 3 weeks, and significantly highest mean bone regeneration (2.80 ± 0.45) and median (3) after 5 weeks, suggesting superior bone regeneration compared to both the control and CM groups. Moreover, the CM group showed a significantly higher mean bone regeneration than the control group at both the three-week interval (1.40 ± 0.55) and five-week interval (2.40 ± 0.55).

Finally, it can be concluded that the CF group consistently showed better results in terms of lower inflammation, absence of fibrosis, and higher bone regeneration. The CM group also performed better than the control group, particularly in the absence of fibrosis at the five-week duration, but the CF group had the most consistently positive outcomes across the parameters measured.

## 4. Discussion

In the current study, two types of chitosan scaffolds were used as scaffolds for bone regeneration, chitosan foam (CF) and chitosan membrane (CM). Both chitosan scaffolds were fabricated by the same fabrication method (freeze-drying), but CM was obtained by an extra final step of the physical crosslinking of chitosan foam using a sodium bicarbonate solution, to obtain the membrane form of chitosan [16].

Recently, the physical crosslinking of chitosan scaffold had gained interest rather than chemical crosslinking due to its lower cost and higher safety [17]. One of the physical crosslinking methods is a neutralization process by direct or gradual exposure to alkaline solutions such as sodium hydroxide or sodium bicarbonate [16]. An increase in pH encourages chitosan to lose its charge, as the pH increases to approach the pKa value of the NH^+3^ groups, which would favor the formation of hydrogen bonding and increasing in chain flexibility to induce gelation [16,18].

Ernesto et al. [19] fabricated chitosan dressings by a freeze-drying process and physical crosslinking by sodium bicarbonate. The resultant chitosan samples showed interconnected porous structures with the ability to adsorb fluid by capacity above 900%, with the favorable cell adhesion and proliferation of fibroblast cells. Accordingly, the physical crosslinking by sodium bicarbonate used in the current study to fabricate chitosan membrane (CM) could explain the small pore size observed by the SEM scanning of CM specimens (mean of 6.4 µm).

A porous chitosan scaffold for bone tissue engineering (BTE) is essential for bone regeneration. In the field of tissue engineering, some works of literature considered that pores with a diameter < 75 µm are micropores, and those >75 µm are macropores [20]. Many research studies have demonstrated the advantage of bone scaffold microporosity regarding protein adsorption and cellular interactions. Microporosity also creates nucleation sites for cellular mineralization, which enhances the osteogenic effect of chitosan scaffolds [8,20]. The previous findings could explain why the chitosan membrane (CM) in the current study showed the highest mesenchymal stem cell proliferation rate in the in vitro testing of the MTT assay after 72 h, due to the microporous nature of the CM scaffold (6.4 µm). Microporosity offers a higher surface area of the scaffold compared to macroporosity, which means a higher osteogenic-related protein adsorption, resulting in a better proliferation, attachment, and differentiation of cells [21].

According to the literature, micropores with a size of >50 µm offer better cell proliferation and attachment than those with a size of <50 µm, while those with size of >50 µm offer better oxygenation and better osteoblast proliferation, as the size of the osteoblast cell is in the range of 10–50 µm [22,23]. Micropores < 50 µm could lead to hypoxic media which favors chondrogenesis rather than osteogenesis [23]. That might explain the better in vivo bone regeneration ability of chitosan foam (CF) compared to that of CM in the current study, which could be explained by the higher mean pore size of CF (65.4 µm) than that of CM (6.4 µm), which might lead to higher in vivo osteoblast proliferation.

On the other side, the macroporosity of chitosan scaffolds enhances vascularization, nutrient supply, and tissue growth [6]. Larger pores support the formation of blood vessels for vascularization within the scaffold. This vascularization is vital for supplying nutrients and removing waste products, further enhancing bone regeneration. Smaller pores are less conducive to blood vessel formation. In vivo, the formation of blood vessels (vascularization) is crucial for supplying nutrients, oxygenation, and removing waste products. The literature had demonstrated that chitosan scaffolds with pores in the range of 100–400 µm were ideal for bone formation and vascularization [8]. Larger pores facilitate better vascularization, which is essential for sustained tissue regeneration [24]. Accordingly, the larger pores of CF might facilitate the better diffusion of nutrients and oxygen, which were essential for bone regeneration [25], with a lower inflammation score than CM, as evident from the histopathological scoring in the current study. Moreover, the higher mean pore size of CF (65.4 µm) could explain the higher vascularity, and copious neovascularization activity, which was observed in the Masson trichome staining of the CF subgroup specimens at the three-week period.

In vitro conditions often have a controlled and abundant supply of nutrients and efficient waste removal. Smaller pores (like 6.4 μm of CM) can support a higher cell density and proliferation because the cells would be in close proximity, facilitating cell–cell interactions and signaling, which explained the high cell proliferation in the case of the MTT assay of the CM group, especially after 72 h [26]. However, in vivo, the nutrient and waste exchange are more complex and rely heavily on vascularization, which might be better supported by the larger pores (65.4 µm) of CF scaffolds [3].

The chitosan foam used in the current study showed a wide range of pore size measurements from >100 µm to <50 µm, with a mean pore size of 65.4 µm. Accordingly, CF possessed the advantages of both micro- and macroporosity in one scaffold, which could explain the higher bone regeneration capacity of CF, while the pores size range of CM was limited to 2.4 to 16.7 µm, which was a microporosity range < 50 µm, that could lead to less than optimal oxygenation and osteoblast proliferation [22,23].

The bone regeneration process may be compromised in patients with diabetes mellitus [27] or hyperlipidemia [28]. According to the findings of the current study, freeze-dried chitosan foam may be promising as a scaffold used in critical-size bone defects for diabetic or hyperlipidemic cases, as it showed a higher bone regeneration capacity in diabetic and hyperlipidemic rats than that of chitosan membrane. We must take into consideration the properties of the chitosan that was used in the current study (medium molecular weight, and 90–95% deacetylation), and the presurgical disinfection method (1 h UV irradiation), as these parameters could have significant effects on the final properties of the fabricated chitosan foam, e.g., porosity, mechanical properties, and degree of crystallinity and degradability [12,29,30,31].

The current study demonstrated how a difference in the treatment method of two scaffolds with the same chemical composition could result in different morphological and architecture properties between them, which resulted in a significant difference in the bone regeneration capacity. Accordingly, the null hypothesis was rejected by the authors.

Controlling the architecture and mechanical properties of the bone scaffold could alter the cellular response, according to the induction of different cellular mechanosignalling pathways. Altering the mechanical stimulus induced from the scaffold could result in improved bone regeneration without the need for growth factors or the seeding of cells [32].

One of the most important limitations in the current study could be related to the requirements of scaffolds for bone regeneration in the maxillofacial region, which are different from those used in other skeletal bones, regarding porosity and other architecture properties [26]. Accordingly, further research studies are needed to evaluate the use of freeze-dried chitosan foam as a scaffold for mandibular bone defects, to evaluate the biocompatibility, degradability, mineralization capacity, and the quality of regenerated bone.

Other properties of the chitosan foam scaffold are needed to be evaluated in future studies, such as biodegradation, compressive strength, and protein adsorption [33].

The current study concentrated mainly on the quality of regenerated bone using CM and CF scaffolds. However, more research studies are needed in the future for a more comprehensive evaluation of the osteoinductive and osteogenic properties of the CF scaffold, such as evaluating the osteogenic differentiation of mesenchymal cells by Alizarin red staining, the expression of Alkaline phosphatase, or an immunohistological examination of OCN and RUNX2 proteins [2,34].

## 5. Conclusions

Within the limitations of this study, the following can be concluded:

1. Both chitosan membrane and chitosan foam scaffolds showed a high bone regeneration capacity in the critical bone defect of rats’ femur bone, in comparison to bone regeneration without using a scaffold.
2. The chitosan foam scaffold showed a significantly higher bone regenerating capacity in critical bone defects of rats’ femur bone, in comparison to the chitosan membrane one.
3. The higher bone regenerating capacity of the chitosan foam scaffold could be related to its higher mean pore size than that of chitosan membrane, in addition to the wide range of chitosan foam porosity (<50 to >100 µm).

## Figures and Tables

**Table 1:** Pore size in CF and CM scaffolds, and comparison between them using Mann–Whitney test (µm).

	Minimum	Maximum	Median	Mean	Std. Deviation	*p* Value
CM	2.40	16.72	5.831	6.44	2.78	<0.0001 *
CF	18.42	184.10	58.63	65.42	31.13	

* Significant difference as *p* ≤ 0.05.

**Table 2:** Optical density of positive control, CM, and CF at T1, T2, and T3.

Duration	Control			CM			CF			*p* Value
	Mean O.D.	SD	Mean Viability %	Mean O.D.	SD	Mean Viability %	Mean O.D.	SD	Mean Viability %	
T1	0.47 ^aA^	0.06	100	1.17 ^aB^	0.12	248.94	1.76 ^aC^	0.06	374.468	0.0001 *
T2	0.62 ^aA^	0.06	100	1.82 ^bB^	0.02	293.55	1.87 ^aB^	0.11	301.6129	0.0001 *
T3	0.81 ^aA^	0.07	100	3.06 ^cB^	0.2	377.78	1.72 ^aC^	0.06	212.3457	0.0001 *
*p* value	0.06			0.007 *			0.29			

^aA^: Means with different superscript letters (small per column/capital per row) were significantly different as *p* < 0.05. * Significant difference as *p* ≤ 0.05.

**Table 3:** Comparison between groups according to histopathological score.

Parameters	Time		Control Group	CM Group	CF Group	Test Value	*p*-Value
Inflammation	3 wks.	Mean ± SD	2.60 ± 0.55 ^A^	2.00 ± 0.71 ^B^	1.00 ± 0.71 ^C^	7.538	0.008
		Median (IQR)	3 (2–3)	2 (2–3)	1 (0–2)		
		Range	2–3	1–3	0–2		
	5 wks.	Mean ± SD	2.20 ± 0.45 ^A^	1.20 ± 0.84 ^B^	0.00 ± 0.00 ^C^	20.222	0.001
		Median (IQR)	2 (2–3)	1 (0–2)	0 (0–0)		
		Range	2–3	0–2	0–0		
Absence of fibrosis	3 wks.	Absent	0 (0.0%)	2 (40.0%)	3 (60.0%)	4.200	0.122
		Present	5 (100.0%)	3 (60.0%)	2 (40.0%)		
	5 wks.	Absent	2 (40.0%)	5 (100.0%)	5 (100.0%)	7.500	0.024
		Present	3 (60.0%) ^A^	0 (0.0%) ^B^	0 (0.0%) ^B^		
Bone vitality	3 wks.	Present	5 (100.0%)	5 (100.0%)	5 (100.0%)	0.000	1.000
		Absent	0 (0.0%)	0 (0.0%)	0 (0.0%)		
	5 wks.	Present	5 (100.0%)	5 (100.0%)	5 (100.0%)	0.000	1.000
		Absent	0 (0.0%)	0 (0.0%)	0 (0.0%)		
Bone regeneration	3 wks.	Mean ± SD	1.00 ± 0.00	1.40 ± 0.55	2.20 ± 0.45	11.200	0.002
		Median (IQR)	1 (1–1) ^C^	1 (1–2) ^B^	2 (2–3) ^A^		
		Range	1–1	1–2	2–3		
	5 wks.	Mean ± SD	1.60 ± 0.55	2.40 ± 0.55	2.80 ± 0.45	7.000	0.010
		Median (IQR)	2 (1–2) ^C^	2 (2–3) ^B^	3 (2–3) ^A^		
		Range	1–2	2–3	2–3		

SD: standard deviation; IQR: interquartile range. Kruskal–Wallis was performed for median (IQR) and multiple comparison between groups through Dunn’s test. Different capital letters indicate significant difference at (*p* < 0.05) among means in the same row.

## Data Availability

All data related to histopathological analysis are available upon request from the authors.

## References

[1] Levengood S.L., Zhang M. (2014). Chitosan-based scaffolds for bone tissue engineering. J. Mater. Chem. B.

[2] Ke Y., Ye Y., Wu J., Ma Y., Fang Y., Jiang F., Yu J. (2023). Phosphoserine-loaded chitosan membranes promote bone regeneration by activating endogenous stem cells. Front. Bioeng. Biotechnol..

[3] Mukasheva F., Adilova L., Dyussenbinov A., Yernaimanova B., Abilev M., Akilbekova D. (2024). Optimizing scaffold pore size for tissue engineering: Insights across various tissue types. Front. Bioeng. Biotechnol..

[4] Cooper D.M.L., Thomas C.D.L., Clement J.G., Turinsky A.L., Sensen C.W., Hallgrímsson B. (2007). Age-dependent change in the 3D structure of cortical porosity at the human femoral midshaft. Bone.

[5] Alonzo M., Alvarez Primo F., Anil Kumar S., Mudloff J.A., Dominguez E., Fregoso G., Ortiz N., Weiss W.M., Joddar B. (2021). Bone tissue engineering techniques, advances, and scaffolds for treatment of bone defects. Curr. Opin. Biomed. Eng..

[6] Yadav P., Beniwal G., Saxena K.K. (2021). A review on pore and porosity in tissue engineering. Mater. Today Proc..

[7] Benedict Terkula Iber Nor Azman Kasan D.T.J.W.O. (2022). A Review of Various Sources of Chitin and Chitosan in Nature. J. Renew. Mater..

[8] Lekhavadhani S., Shanmugavadivu A., Selvamurugan N. (2023). Role and architectural significance of porous chitosan-based scaffolds in bone tissue engineering. Int. J. Biol. Macromol..

[9] Grzybek P., Jakubski Ł., Dudek G. (2022). Neat Chitosan Porous Materials: A Review of Preparation, Structure Characterization and Application. Int. J. Mol. Sci..

[10] Medhat A., El-Zainy M.A., Fathy I. (2024). Photo biomodulation of dental derived stem cells to ameliorate regenerative capacity: In vitro study. Saudi Dent. J..

[11] Liu X., Wang Z. (2023). Chitosan-calcium carbonate scaffold with high mineral content and hierarchical structure for bone regeneration. Smart Mater. Med..

[12] Dai Z., Ronholm J., Tian Y., Sethi B., Cao X. (2016). Sterilization techniques for biodegradable scaffolds in tissue engineering applications. J. Tissue Eng..

[13] Ezoddini-Ardakani F., Navabazam A., Fatehi F., Danesh-Ardekani M., Khadem S., Rouhi G. (2012). Histologic evaluation of chitosan as an accelerator of bone regeneration in microdrilled rat tibias. Dent. Res. J..

[14] De la Riva B., Sánchez E., Hernández A., Reyes R., Tamimi F., López-Cabarcos E., Delgado A., Evora C. (2010). Local controlled release of VEGF and PDGF from a combined brushite-chitosan system enhances bone regeneration. J. Control. Release.

[15] Poser L., Matthys R., Schawalder P., Pearce S., Alini M., Zeiter S. (2014). A standardized critical size defect model in normal and osteoporotic rats to evaluate bone tissue engineered constructs. Biomed. Res. Int..

[16] Fletes-Vargas G., Espinosa-Andrews H., Cervantes-Uc J.M., Limón-Rocha I., Luna-Bárcenas G., Vázquez-Lepe M., Morales-Hernández N., Jiménez-Ávalos J.A., Mejía-Torres D.G., Ramos-Martínez P. (2023). Porous Chitosan Hydrogels Produced by Physical Crosslinking: Physicochemical, Structural, and Cytotoxic Properties. Polymers.

[17] Li P., Zhao J., Chen Y., Cheng B., Yu Z., Zhao Y., Yan X., Tong Z., Jin S. (2017). Preparation and characterization of chitosan physical hydrogels with enhanced mechanical and antibacterial properties. Carbohydr. Polym..

[18] Rivas-Araiza R., Alcouffe P., Rochas C., Montembault A., David L. (2010). Micron Range Morphology of Physical Chitosan Hydrogels. Langmuir.

[19] Ernesto J.V., Gasparini Í., Corazza F., Mathor M., Silva C., Leite-Silva V., Andreo-Filho N., Lopes P. (2023). Physical, chemical, and biological characterization of biodegradable chitosan dressing for biomedical applications: Could sodium bicarbonate act as a crosslinking agent?. Mater. Chem. Phys..

[20] Perez R.A., Mestres G. (2016). Role of pore size and morphology in musculo-skeletal tissue regeneration. Mater. Sci. Eng. C.

[21] Zhang K., Fan Y., Dunne N., Li X. (2018). Effect of microporosity on scaffolds for bone tissue engineering. Regen. Biomater..

[22] Sugawara Y., Kamioka H., Honjo T., Tezuka K., Takano-Yamamoto T. (2005). Three-dimensional reconstruction of chick calvarial osteocytes and their cell processes using confocal microscopy. Bone.

[23] Lin T.-H., Wang H.-C., Cheng W.-H., Hsu H.-C., Yeh M.-L. (2019). Osteochondral Tissue Regeneration Using a Tyramine-Modified Bilayered PLGA Scaffold Combined with Articular Chondrocytes in a Porcine Model. Int. J. Mol. Sci..

[24] Zhang Q., Yuan C., Liu L., Wen S., Wang X. (2022). Effect of 3-dimensional Collagen Fibrous Scaffolds with Different Pore Sizes on Pulp Regeneration. J. Endod..

[25] Piszko P.J., Piszko A., Kiryk S., Kiryk J., Horodniczy T., Struzik N., Wiśniewska K., Matys J., Dobrzyński M. (2024). Bone Regeneration Capabilities of Scaffolds Containing Chitosan and Nanometric Hydroxyapatite—Systematic Review Based on In Vivo Examinations. Biomimetics.

[26] Huang X., Lou Y., Duan Y., Liu H., Tian J., Shen Y., Wei X. (2024). Biomaterial scaffolds in maxillofacial bone tissue engineering: A review of recent advances. Bioact. Mater..

[27] Cai F., Liu Y., Liu K., Zhao R., Chen W., Yusufu A., Liu Y. (2023). Diabetes mellitus impairs bone regeneration and biomechanics. J. Orthop. Surg. Res..

[28] Shang J., Li Z., Ma A., Zhu T., Ma G., Gui H., Ren H., Sun B., Wang W., Wang X. (2025). Hyperlipidemia impairs bone repair and regeneration via miR-193a-3p/STMN1/PI3K/Akt axis. Biochem. Pharmacol..

[29] Nwe N., Furuike T., Tamura H. (2009). The Mechanical and Biological Properties of Chitosan Scaffolds for Tissue Regeneration Templates Are Significantly Enhanced by Chitosan from *Gongronella butleri*. Materials.

[30] Tangsadthakun C., Kanokpanont S., Sanchavanakit N., Pichyangkura R., Banaprasert T., Tabata Y., Damrongsakkul S. (2007). The influence of molecular weight of chitosan on the physical and biological properties of collagen/chitosan scaffolds. J. Biomater. Sci. Polym. Ed..

[31] Balagangadharan K., Dhivya S., Selvamurugan N. (2017). Chitosan based nanofibers in bone tissue engineering. Int. J. Biol. Macromol..

[32] Rajendran A.K., Sankar D., Amirthalingam S., Kim H.D. (2023). Trends in mechanobiology guided tissue engineering and tools to study cell-substrate interactions: A brief review. Biomater. Res..

[33] Venkatesan J., Bhatnagar I., Kim S.-K. (2014). Chitosan-alginate biocomposite containing fucoidan for bone tissue engineering. Mar. Drugs.

[34] Abudukelimu K., Aierken A., Tuerxuntayi A., Yilihamu Y., Abulizi S., Wufuer D., Dong H. (2024). Preliminary study on the preparation of antler powder/chitosan/β-glycerophosphate sodium/polyvinyl alcohol porous hydrogel scaffolds and their osteogenic effects. Front. Bioeng. Biotechnol..

